# Cardiopulmonary exercise testing (CPET) as preoperative test before lung resection

**DOI:** 10.1186/1939-4551-8-S1-A22

**Published:** 2015-04-08

**Authors:** Kostantinos Syrigos, Anastasios Kallianos, Aggeliki Rapti, Sotirios Tsimpoukis, Andriani Charpidou, Ioannis Ntanos, Elias Kainis

**Affiliations:** 1 Sotiria General Hospital, Athens School of Medicine, Greece

## Background

Lung resection is still the only potentially curative therapy for patients with localised non-small lung cancer (NSCLC). However, the presence of cardiovascular comorbidities and underlying lung disease increases the risk of postoperative complications. Various studies have evaluated the use of different preoperative tests in order to identify patients with an increased risk for postoperative complications, associated with prolonged hospital stay and increased morbidity and mortality.

## Methods

In this topic review, we discuss the role of cardiopulmonary exercise testing (CPET) as one of the preoperative tests suggested for lung cancer patients scheduled for lung resection. We describe different types of exercise testing techniques and present algorithms of preoperative evaluation in lung cancer patients.

## Results

Patients without known underlying lung disease with a preoperative FEV1 (forced expiratory volume in one second) greater than 2 L generally tolerate well pneumonectomy, whereas those with FEV1 greater than 1.5 L are expected to tolerate lobectomy. Although spirometric values strongly correlate with the severity of obstruction, they do not provide direct information regarding the degree of gas exchange and cardiovascular reserve. CPET reflects interactions between pulmonary function, cardiovascular status and oxygen uptake and utilization by the peripheral tissues.

## Conclusions

Overall, patients with maximal oxygen consumption (VO_2_ max) <10 mL/kg/min or those with VO_2_ max <15 mL/kg/min and both postoperative FEV1 and DLCO <40% predicted are at high risk for perioperative death and postoperative cardiopulmonary complications, and thus should be offered an alternative medical treatment option.

